# Impact of combining laparoscopy with traditional Chinese medicine on oxidative stress in endometriosis-related infertility: A systematic review and meta-analysis

**DOI:** 10.1097/MD.0000000000041692

**Published:** 2025-04-11

**Authors:** Shaoqiao Lin, Shanshan Zhou

**Affiliations:** a Community Health Service Center, Wanqingsha Town, Nansha District, Guangzhou City, Guangdong Province, China; b Zhuhai Xiangzhou District People’s Hospital, Zhuhai City, Guangdong Province, China.

**Keywords:** endometriosis-related infertility, oxidative stress, traditional Chinese medicine

## Abstract

**Background:**

To evaluate the effect of laparoscopic procedures integrated with traditional Chinese medicine (TCM), specifically aimed at enhancing blood flow and alleviating blood stasis, on oxidative stress levels in individuals with endometriosis-induced infertility.

**Methods:**

We performed a systematic quantitative review to evaluate the effects of laparoscopic surgery combined with TCM on oxidative stress in endometriosis-related infertility by enhancing blood circulation and resolving stasis. A literature search of 9 major databases was performed. Meta-analysis was performed using Review Manager version 5.4 (R Foundation for Statistical Computing, Vienna, Austria) and Stata Release 16.0 (StataCorp LLC, College Station, TX). This systematic review was registered with the International Prospective Register of Systematic Reviews (i.e., “PROSPERO”) (registration number: CRD42024526800).

**Results:**

Thirteen trials comprising 1084 participants were included. Laparoscopy combined with TCM for promoting blood circulation and removing blood stasis significantly reduced the levels of 8-isoprostane prostaglandin F2α (mean difference [MD] −29.57 [95% confidence interval (CI) −32.65 to −26.49]; *P* < .001), regulated on activation, normal T cell expressed and secreted (MD −231.83 [95% CI −341.33 to −122.32]; *P* < .001), reactive oxygen species (MD −0.92; [95% CI −1.12 to −0.73]; *P* < .001), monocyte chemoattractant protein-1 (MD −31.23 [95% CI −42.70 to −19.77]; *P* < .001), and increased glutathione peroxidase (MD 31.45 [95% CI 26.04 to 36.87]; *P* < .001), vitamin E (MD 4.86 [95% CI 3.77 to 5.94]; *P <* .001), superoxide dismutase (standardized MD 0.92 [95% CI 0.41 to 1.42]; *P* < .001).

**Conclusion:**

Compared with laparoscopic surgery alone, the combination of TCM for promoting blood circulation and removing blood stasis demonstrated the potential to ameliorate oxidative stress in patients with endometriosis-induced infertility. However, further large-scale clinical trials are required to confirm these findings.

## 1. Introduction

Endometriosis, a prevalent gynecological ailment, is characterized by abnormal proliferation of endometrial tissue at extrauterine sites and can lead to chronic inflammation, pelvic mass formation, pelvic pain, and infertility.^[1–3]^ Clinically, endometriosis affects an estimated 10% to 15% of women globally, and 35% to 50% of those affected experience infertility.^[4,5]^ Endometriosis-related infertility affects not only women’s physical health and well-being, but also their marital and family relationships, leading to physical and emotional distress.^[6]^

Oxidative stress is triggered by an imbalance in which the production of pro-oxidants outweighs the availability of antioxidants, leading to an overabundance of reactive oxygen species (ROS) that the body cannot sufficiently counteract.^[7]^ Recent studies have demonstrated a correlation between endometriosis and oxidative stress levels,^[8,9]^ with increased stress being associated with a higher risk for this condition.^[10]^ Research suggests that oxidative stress can worsen pelvic adhesions in individuals with endometriosis.^[11]^ Furthermore, oxidative stress-induced DNA damage is considered to be a crucial contributor to endometriosis-associated infertility.^[12]^ Laparoscopic surgery is an important therapeutic approach for endometriosis-associated infertility. However, laparoscopic surgery often poses challenges, such as low pregnancy rates and high postoperative recurrence, and continuous medication is required even after surgery. New therapies, including antioxidants, may partially counteract ectopic endometriosis lesions, whereas steroids and tumor necrosis factor-α antagonists may modulate immune factors associated with endometriosis. However, clinical evidence supports these treatments.^[13]^ Currently, antioxidant therapy for patients with endometriosis remains in the preclinical animal testing phase and additional clinical trials are needed to validate its efficacy.^[14]^

Traditional Chinese medicine (TCM) posits that endometriosis-related infertility is associated with blood stasis. In “The Source of All Diseases and Syndromes of Women: Miscellaneous Diseases,” it is mentioned that blood mass diseases, caused by blood aggregation, result in symptoms such as lumbago and limited mobility. Conditions, such as acute lower abdominal pain, spinal soreness, and deep pelvic cramping, are associated with what is termed “childless disease,” which presents as pelvic pain, irregular menstruation, infertility, and symptoms similar to those of endometriosis. The principal therapeutic approach focuses on enhancing blood flow and dissipating blood stasis. Emerging research has indicated the potential of integrating laparoscopic techniques with TCM strategies for blood flow improvement and stasis alleviation to mitigate the oxidative stress typically encountered in endometriosis. However, the current evidence is insufficient to draw definitive conclusions. As such, this meta-analysis evaluated the effect of TCM, aimed at enhancing blood flow and dissipating blood stasis, on oxidative stress levels in individuals with endometriosis-related infertility. The aim was to gather compelling scientific data to inform clinical decision making.

## 2. Methods

The present meta-analysis analyzed data published in the public domain and did not involve any new data collection or experimental procedures. As such, it was not subject to ethics review in accordance with the guidelines of the authors’ institution.

### 2.1. Inclusion and exclusion criteria

Studies that fulfilled the following criteria were considered to be eligible for inclusion: randomized controlled trials (RCTs), encompassing both double- and non-double-blind designs, as well as case–control studies; patients diagnosed with endometriosis-related infertility, who did not receive clinical treatment; the experimental group underwent laparoscopy and TCM for circulation and stasis, whereas the control group underwent laparoscopy with or without Western medicine; and included ≥ 1 outcome measure(s), including glutathione peroxidase (GSH-Px), monocyte chemoattractant protein-1 (MCP-1), regulated on activation, normal T cell expressed and secreted (RANTES), 8-isoprostane prostaglandin F2α (8-ios-PGF2α), ROS, vitamin E (Vit E), and superoxide dismutase (SOD).

The exclusion criteria were as follows: non-infertile patients; studies lacking laboratory indicators of oxidative stress, and cases in which the subjects and interventions failed to fulfill the inclusion criteria; incomplete or incorrect data; those from which the full text could not be obtained, and those that lacked relevant outcome indicators; and duplicate studies, reviews, case reports, animal studies, and nonclinical STU studies.

### 2.2. Search strategy

**Figure 1.**
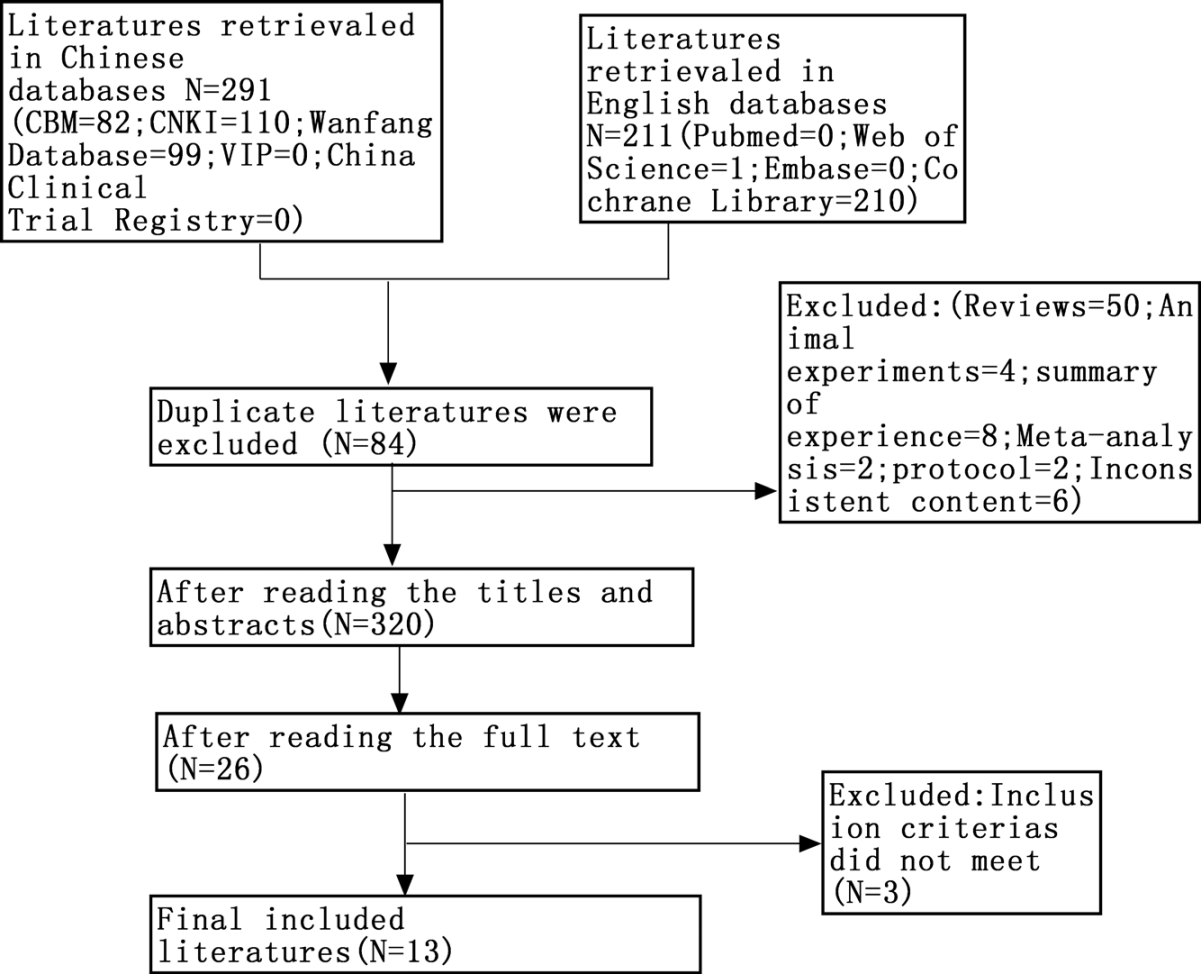
PRISMA flow diagram of study selection.

One author systematically searched multiple databases, including PubMed, Embase, Cochrane Library, Web of Science, Chinese National Knowledge Infrastructure (i.e., “CNKI”), Wanfang, Chinese Scientific Journal Database (i.e., “VIP”), and Chinese Biomedical Database (i.e., “CBM”), from their inception to March 2, 2024 for relevant studies. Furthermore, the Chinese Clinical Trial Registry was consulted to identify eligible, ongoing, and/or unpublished trials. The search parameters were restricted to articles published in either Chinese or English. The search strategy is illustrated in Figure 1, with detailed strategies provided in Table 1 and Table S1, Supplemental Digital Content, http://links.lww.com/MD/O442.

**Table 1:** Search strategy.

Pubmed		
1	“Endometriosis” [MeSH Terms]	26,362
2	“Endometrioses” [Title/Abstract] OR “Endometrioma” [Title/Abstract] OR “Endometriomas” [Title/Abstract]	3240
3	“Endometrioses” [Title/Abstract] OR “Endometrioma” [Title/Abstract] OR “Endometriomas” [Title/Abstract] OR “Endometriosis” [MeSH Terms]	27,147
4	“Infertility” [MeSH Terms]	75,218
5	“Infertility” [Title/Abstract] OR “Sterility” [Title/Abstract] OR “reproductive sterility” [Title/Abstract] OR “Subfertility” [Title/Abstract] OR “Sub-Fertility” [Title/Abstract]	90,554
6	“Infertility” [MeSH Terms] OR “Infertility” [Title/Abstract] OR “Sterility” [Title/Abstract] OR “reproductive sterility” [Title/Abstract] OR “Subfertility” [Title/Abstract] OR “Sub-Fertility” [Title/Abstract]	123,321
7	(“activating blood circulation” [Title/Abstract] AND “removing blood stasis” [Title/Abstract]) OR “huoxuehuayu” [Title/Abstract] OR “huoxue” [Title/Abstract]	590
8	“Laparoscopy” [MeSH Terms]	121,271
9	“Laparoscopies” [Title/Abstract] OR “Celioscopy” [Title/Abstract] OR “Celioscopies” [Title/Abstract] OR “Peritoneoscopy” [Title/Abstract] OR “Peritoneoscopies” [Title/Abstract] OR “laparoscopic surgical procedure” [Title/Abstract] OR “laparoscopic surgical procedures” [Title/Abstract] OR “laparoscopic surgery” [Title/Abstract] OR “laparoscopic surgeries” [Title/Abstract] OR “laparoscopic assisted surgery” [Title/Abstract] OR “laparoscopic assisted surgeries” [Title/Abstract]	27,162
10	“Laparoscopies” [Title/Abstract] OR “Celioscopy” [Title/Abstract] OR “Celioscopies” [Title/Abstract] OR “Peritoneoscopy” [Title/Abstract] OR “Peritoneoscopies” [Title/Abstract] OR “laparoscopic surgical procedure” [Title/Abstract] OR “laparoscopic surgical procedures” [Title/Abstract] OR “laparoscopic surgery” [Title/Abstract] OR “laparoscopic surgeries” [Title/Abstract] OR “laparoscopic assisted surgery” [Title/Abstract] OR “laparoscopic assisted surgeries” [Title/Abstract] OR “Laparoscopy” [MeSH Terms]	129,780
11	(“Endometrioses” [Title/Abstract] OR “Endometrioma” [Title/Abstract] OR “Endometriomas” [Title/Abstract] OR “Endometriosis” [MeSH Terms]) AND (“Infertility” [MeSH Terms] OR (“Infertility” [Title/Abstract] OR “Sterility” [Title/Abstract] OR “reproductive sterility” [Title/Abstract] OR “Subfertility” [Title/Abstract] OR “Sub-Fertility” [Title/Abstract])) AND ((“activating blood circulation” [Title/Abstract] AND “removing blood stasis” [Title/Abstract]) OR “huoxuehuayu” [Title/Abstract] OR “huoxue” [Title/Abstract]) AND (“Laparoscopies” [Title/Abstract] OR “Celioscopy” [Title/Abstract] OR “Celioscopies” [Title/Abstract] OR “Peritoneoscopy” [Title/Abstract] OR “Peritoneoscopies” [Title/Abstract] OR “laparoscopic surgical procedure” [Title/Abstract] OR “laparoscopic surgical procedures” [Title/Abstract] OR “laparoscopic surgery” [Title/Abstract] OR “laparoscopic surgeries” [Title/Abstract] OR “laparoscopic assisted surgery” [Title/Abstract] OR “laparoscopic assisted surgeries” [Title/Abstract] OR “Laparoscopy” [MeSH Terms])	0
Embase		
1	“endometriosis”/exp OR endometriosis	57,481
2	“Endometrioses”:ab,ti OR “Endometrioma”:ab,ti OR “Endometriomas”:ab,ti	5206
3	#1 OR #2	58,638
4	“infertility”/exp OR infertility	220,318
5	“infertility”:ab,ti OR “sterility”:ab,ti OR “reproductive sterility”:ab,ti OR “subfertility”:ab,ti OR “sub-fertility”:ab,ti	124,141
6	#4 OR #5	234,499
7	“activating blood circulation and removing blood stasis”:ab,ti OR “huoxuehuayu”:ab,ti OR “huoxue”:ab,ti	869
8	“laparoscopic” OR laparoscopic	262,781
9	“laparoscopies”:ab,ti OR “celioscopy”:ab,ti OR “celioscopies”:ab,ti OR “peritoneoscopy”:ab,ti OR “peritoneoscopies”:ab,ti OR “laparoscopic surgical procedure”:ab,ti OR “laparoscopic surgical procedures”:ab,ti OR “laparoscopic surgery”:ab,ti OR “laparoscopic surgeries”:ab,ti OR “laparoscopic assisted surgery”:ab,ti OR “laparoscopic assisted surgeries”:ab,ti	40,083
10	#8 OR #9	264,586
11	#3 OR #6 OR #10	0
Web of Science		
1	TS = (Endometriosis OR Endometrioses OR Endometrioma OR Endometriomas)	46,943
2	TS = (Infertility OR Sterility OR Reproductive Sterility OR Subfertility OR Sub-Fertility)	198,299
3	TS = (Activating blood circulation and removing blood stasis OR huoxuehuayu OR huoxue)	3252
4	TS = (laparoscopic OR Laparoscopies OR Celioscopy OR Celioscopies OR Peritoneoscopy OR Peritoneoscopies OR Laparoscopic Surgical Procedure OR Laparoscopic Surgical Procedures OR Laparoscopic Surgery OR Laparoscopic Surgeries OR Laparoscopic Assisted Surgery OR Laparoscopic Assisted Surgeries)	217,022
5	#1 AND #2 AND #3 AND #4	1
Cochrane		
1	Endometriosis	3427
2	(Endometrioses):ab,ti,kw OR (Endometrioma):ab,ti,kw OR (Endometriomas):ab,ti,kw	429
3	#1 OR #2	3540
4	Infertility	12,615
5	(Infertility):ab,ti,kw OR (Sterility):ab,ti,kw OR (Reproductive Sterility):ab,ti,kw OR (Subfertility):ab,ti,kw OR (Sub-Fertility):ab,ti,kw	12,146
6	#4 OR #5	13,319
7	laparoscopic	26,870
8	(Laparoscopies):ab,ti,kw OR (Celioscopy):ab,ti,kw OR (Celioscopies):ab,ti,kw OR (Peritoneoscopy):ab,ti,kw OR (Peritoneoscopies):ab,ti,kw OR (Laparoscopic Surgical Procedure):ab,ti,kw OR (Laparoscopic Surgical Procedures):ab,ti,kw OR (Laparoscopic Surgery):ab,ti,kw OR (Laparoscopic Surgeries):ab,ti,kw OR (Laparoscopic Assisted Surgery):ab,ti,kw OR (Laparoscopic Assisted Surgeries):ab,ti,kw	21,401
9	#7 OR #8	26,924
10	#3 AND #6 AND #9	210
CBM		
1	“Endometriosis”[Mesh Terms]	22,549
2	“ Endometrioses “[Commonly used fields: intelligence]” OR “ Endometrial cyst” “[Commonly used fields: intelligence]” OR “ Endometriosis”[Commonly used fields: intelligence] OR “ Chocolate cyst “[Commonly used fields: intelligence] OR “ Ovarian endometriosis cyst “[Commonly used fields: intelligence]	27,719
3	(#2) OR (#1)	27,719
4	“Infertility, female”[Mesh Terms]	16,414
5	“ infertility “[Commonly used fields: intelligence] OR “ sterility “[Commonly used fields: intelligence] OR “ infertile”[Commonly used fields: intelligence] OR “ female infertility “[Commonly used fields: intelligence]	48,136
6	(#4) OR (#5)	49,136
7	“Blood-activating and stasis-removing agent”[Mesh Terms]	102,530
8	“ Blood-activating and stasis-eliminating method “[Commonly used fields: intelligence] OR “ Promoting blood circulation and removing blood stasis “[Commonly used fields: intelligence] OR “ Promoting blood circulation and removing blood stasis “[Commonly used fields: intelligence] OR “ Blood-activating and stasis-removing prescriptions “[Commonly used fields: intelligence] OR “ Chinese herbal medicine for promoting blood circulation and removing blood stasis “[Commonly used fields: intelligence] OR “ blood-activating and stasis-removing drugs “[Commonly used fields: intelligence] OR “ improve blood circulation “[Commonly used fields: intelligence]	163,078
9	(#7) OR (#8)	235,456
10	“ Laparoscopic examination” [Unweighted: Extended]	121,873
11	“Laparoscopy” [common field: intelligence] OR “laparoscopic surgery” [common field: intelligence]	184,266
12	(#10) OR (#11)	184,270
13	(#3) AND (#6) AND (#9) AND (#12)	82
CNKI		
1	“ Endometrioses “[Commonly used fields: intelligence] OR “ Endometrial cyst “[Commonly used fields: intelligence] OR “ Endometriosis”[Commonly used fields: intelligence] OR “ Chocolate cyst “[Commonly used fields: intelligence] OR “ Ovarian endometriosis cyst “[Commonly used fields: intelligence] OR “endometriosis”[Mesh Terms]	30,479
2	“ Infertility “[Commonly used fields: intelligence] OR “ sterility “[Commonly used fields: intelligence] OR “ infertile”[Commonly used fields: intelligence] OR “ female infertility “[Commonly used fields: intelligence] OR “Infertility, female”[Mesh Terms]	50,765
3	“ Blood-activating and stasis-eliminating method “[Commonly used fields: intelligence] OR “ Promoting blood circulation and removing blood stasis “[Commonly used fields: intelligence] OR “ Promoting blood circulation and removing blood stasis “[Commonly used fields: intelligence] OR “ Blood-activating and stasis-removing prescriptions “[Commonly used fields: intelligence] OR “ Chinese herbal medicine for promoting blood circulation and removing blood stasis “[Commonly used fields: intelligence] OR “ blood-activating and stasis-removing drugs “[Commonly used fields: intelligence] OR “ improve blood circulation “[Commonly used fields: intelligence] OR “Blood-activating and stasis-removing agent”[Mesh Terms]	207,460
4	Laparoscopic examination “ [Unweighted: Extended]” OR “Laparoscopy” [common field: intelligence] OR “laparoscopic surgery” [common field: intelligence]	110
Wanfang Database		
1	Theme (“ Endometrioses “ OR “ Endometrial cyst “ OR “ Endometriosis” OR “ Chocolate cyst “ OR “ Ovarian endometriosis cyst “) AND Theme (“ infertility “ OR “ sterility “ OR “ infertile “ OR “ female infertility “ OR “Infertility, female”[Mesh Terms]) AND Theme (“ blood-activating and stasis-eliminating method “ OR “ Promoting blood circulation and removing blood stasis “ OR “ Promoting blood circulation and removing blood stasis “ OR “ Blood-activating and stasis-removing prescriptions “ OR “ Chinese herbal medicine for promoting blood circulation and removing blood stasis “ OR “ blood-activating and stasis-removing drugs “ OR “ improve blood circulation “ OR “Blood-activating and stasis-removing agent”[Mesh Terms])AND Theme (“Laparoscopic examination ” OR “Laparoscopy” OR “laparoscopic surgery”)	99
VIP		
1	Theme (“ Endometrioses “ OR “ Endometrial cyst “ OR “ Endometriosis” OR “ Chocolate cyst “ OR “ Ovarian endometriosis cyst “) AND Theme (“ infertility “ OR “ sterility “ OR “ infertile “ OR “ female infertility “ OR “Infertility, female”[Mesh Terms]) AND Theme (“ blood-activating and stasis-eliminating method “ OR “ Promoting blood circulation and removing blood stasis “ OR “ Promoting blood circulation and removing blood stasis “ OR “ Blood-activating and stasis-removing prescriptions “ OR “ Chinese herbal medicine for promoting blood circulation and removing blood stasis “ OR “ blood-activating and stasis-removing drugs “ OR “ improve blood circulation “ OR “Blood-activating and stasis-removing agent”[Mesh Terms])AND Theme (“Laparoscopic examination ” OR “Laparoscopy” OR “laparoscopic surgery”)	0
Chinese clinical trial registration		
1	Disease name: endometriosis, research type: prevention, Intervention measure: Laparoscopy	0

### 2.3. Literature screening and data extraction

Two researchers independently screened the retrieved studies by reading titles and abstracts, excluding duplicates and those that did not fulfill the inclusion criteria, and thoroughly examined the full texts of eligible studies.^[15–17]^ They cross-checked the results of the included studies, assessed quality, and in the event of disagreement, a third researcher mediated to achieve consensus. The extracted data comprised the following essential study elements: author names and publication year; sample size, participant age, and demographic data; details of interventions for both experimental and control groups; outcome measures, including GSH-Px, MCP-1, RANTES, 8-ios-PGF2α, ROS, Vit E, and SOD (Table 2).

**Table 2:** Information of included studies.

Inclusion study	Age ($\bar{x}\pm s$) years		No. of participants		Intervention measure		Course of treatment (month)	Outcome indexes
	T	C	T	C	T	C		
Zhang Xiaosha (2017)^[18]^	33.36 ± 3.18	32.89 ± 4.0	30	30	Laparoscopic surgery + Chinese medicine for promoting blood circulation and removing blood stasis	Laparoscopic surgery	3	⑦
Zhang Mei (2017)^[19]^	29. 80 ± 3. 65	29. 70 ± 3. 24	35	35	Laparoscopic surgery + Chinese medicine for promoting blood circulation and removing blood stasis and tonifying kidney + Dafilin	Laparoscopic surgery + Dafilin	3	⑦
Chen Xiaoni (2015)^[20]^	27.5± 3.3	27.5 ± 3.8	49	49	Laparoscopic surgery + Chinese medicine for promoting blood circulation and removing blood stasis	Laparoscopic surgery	3	②③④
Wu Shufen (2013)^[21]^	28.5 ± 3.7	29.1 ± 3.9	60	60	Laparoscopic surgery + Chinese medicine for promoting blood circulation and removing blood stasis	Laparoscopic surgery	3	④
Shi Jinmei (2019)^[22]^	28.49 ± 6.28	27.15 ± 5.87	60	60	Laparoscopic surgery + Chinese medicine for promoting blood circulation and removing blood stasis and warming kidney Chinese medicine	Laparoscopic surgery	3	⑤⑥⑦
Yang Ling (2016)^[23]^	26.98 ± 2.31	27.32 ± 2.45	42	42	Laparoscopic surgery + Chinese medicine for promoting blood circulation and removing blood stasis	Laparoscopic surgery	3	⑤⑥⑦
Deng Mingyu (2022)^[24]^	26.89 ± 2.25	26.89 ± 2.25	54	54	Laparoscopic surgery + Chinese medicine for promoting blood circulation and removing blood stasis and tonifying kidney	Laparoscopic surgery	3	⑤⑥
Yang Xuefang (2016)^[25]^	29.4 ± 3.6	29.4 ± 3.6	69	69	Laparoscopic surgery + Chinese medicine for promoting blood circulation and removing blood stasis	Laparoscopic surgery	3	②③④
Xu Qian (2016)^[26]^	33.39 ± 3.20	33.39 ± 3.20	25	25	Laparoscopic surgery + Chinese medicine for promoting blood circulation and removing blood stasis	Laparoscopic surgery	3	④
He Wenjing (2019)^[27]^	27.93 ± 2.04	28.05 ± 2.12	63	63	Laparoscopic surgery + Chinese medicine for promoting blood circulation and removing blood stasis	Laparoscopic surgery	3	⑦
Chen Xiaoping (2013)^[28]^	30. 28 ± 3.169	2. 76 ± 0. 333	17	23	Laparoscopic surgery + Chinese medicine for promoting blood circulation and removing blood stasis	Laparoscopic surgery	3	③
Li Haifang (2012)^[29]^	28.87 ± 3.66	30.27 ± 4.22	15	15	Laparoscopic surgery + Chinese medicine for promoting blood circulation and removing blood stasis	Laparoscopic surgery + GnRH-a	3	②
Luo Meiling (2012)^[30]^	30.28 ± 3.16	31.08 ± 3.54	17	23	Laparoscopic surgery + Chinese medicine for promoting blood circulation and removing blood stasis	Laparoscopic surgery	3	③

T = experimental group; C = control group; ① GSH-Px; ② MCP-1; ③ RANTES; ④ 8-ios-PGF2α; ⑤ ROS; ⑥ Vit E; ⑦ SOD.

GSH-Px = glutathione peroxidase, 8-ios-PGF2α = 8-isoprostane prostaglandin F2α, MCP-1 = monocyte chemoattractant protein-1, RANTES = regulated on activation, normal T cell expressed and secreted, ROS = reactive oxygen species, SOD = superoxide dismutase, Vit E = vitamin E.

### 2.4. Literature quality evaluation

**Figure 2.**
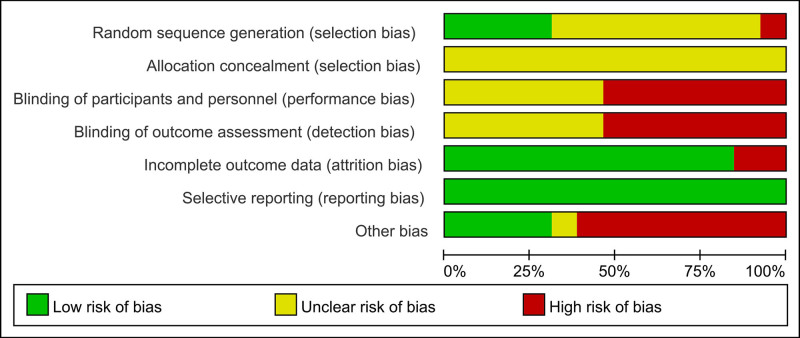
Summary of risk of bias: review of the authors’ judgments on the risk of bias for each of the 13 RCTs included. RCT = randomized controlled trial.

Two researchers independently evaluated the quality of each RCT using the Cochrane Collaboration’s Risk-of-Bias assessment tool, as depicted in Figure 2. This assessment tool addresses the following: random allocation methods; blinding, determining whether the study was blinded for researchers, subjects, and outcome evaluators; integrity of outcome data; selective reporting, identifying any indications of a bias toward reporting only specific outcomes; and other biases. Studies were stratified into 3 risk-of-bias risk categories based on methodological quality: high, low, and unclear. Disagreements were addressed through collaborative discussions and resolutions among the researchers.

### 2.5. Conclusion

8-ios-PGF2α, RANTES, ROS, MCP-1, GSH-Px, Vit E, and SOD.

### 2.6. Statistical analysis

Meta-analysis was performed using Review Manager version 5.3.3 (R Foundation for Statistical Computing, Vienna, Austria), and Stata Release 16.0 (StataCorp LLC, College Station, TX). Relative risk and corresponding 95% confidence interval (95% CI) were calculated for binary variables. Continuous data are expressed as standardized mean difference with corresponding 95% CI. Heterogeneity was assessed using the *Q* test and I² statistic. A *P*-value < .1 or I² value ≥ 50% signified heterogeneity among the study findings. After detecting heterogeneity, the meta-analysis used a random-effects model, and sensitivity and subgroup analyses were performed to identify the sources of heterogeneity. The fixed-effects model was used in the absence of significant heterogeneity.

## 3. Results

### 3.1. Search results

The initial literature search retrieved 502 relevant studies. After a thorough selection process, 13 eligible trials^[18–30]^ were included in the final meta-analysis (Table 2). All studies included in the meta-analysis, referenced as,^[18–30]^ were conducted exclusively in China and comprised 1084 patients. Among the total enrolled participants, 536 were assigned to the experimental group and 548 to the control group, with the studies spanning a period of 3 months. The treatment group underwent laparoscopic procedures combined with TCM designed to improve blood flow and eliminate blood stasis, whereas the control group underwent laparoscopic surgery, either as a standalone treatment or in conjunction with Western medical therapy. A summary of the included studies is presented in Table 2.

### 3.2. Risk of bias

The methodological quality of the studies was appraised using the Cochrane Collaboration’s bias risk assessment. The 13 included studies^[18–30]^ were RCTs. The potential for bias in RCTs was evaluated using the Cochrane Collaboration tool (Fig. 2). Four studies^[19,22,24,27]^ used the random number table method for randomization, 1 study^[20]^ used the odd-even random method, and 8 studies^[18,19,21,23,25,26,28,29]^ acknowledged the necessity of randomization yet omitted the description of the methods used. Importantly, concealment of the allocation scheme details was absent from all 13 studies.^[18–30]^ Seven studies^[18,19,21–24,29]^ reported that the participants provided informed consent. Nonetheless, 6 studies^[20,21,25,26,28,30]^ omitted details of the application of blinding methods and procedures for assessing research outcomes. Despite these limitations, all 13 studies^[18–30]^ specified preestablished outcome measures with no indication of selective reporting, 11 studies^[18–27,29]^ reported complete results, and 2 studies^[28,30]^ presented incomplete data. Four studies^[22,24,25,27]^ showed no evidence of any other bias; however, 8 studies^[18–20,23,26,28–30]^ were identified as having a small sample size bias, and 11 studies^[18–20,22–27,29,30]^ reported no statistically significant differences in baseline data.

### 3.3. Outcomes of meta-analysis

#### 3.3.1. 8-ios-PGF2α

**Figure 3.**
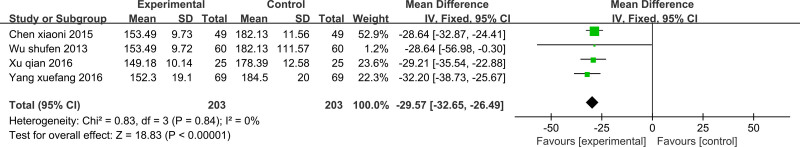
Forest plot of 8-ios-PGF2α: Forest plot of comparing TCM to promote blood circulation and remove blood stasis combined with laparoscopy versus laparoscopy alone on 8-ios-PGF2α in endometriosis-related infertility patients. 8-ios-PGF2α = 8-isoprostane prostaglandin F2α, TCM = traditional Chinese medicine.

8-ios-PGF2α was reported in 4 studies^[20,21,25,26]^; analysis revealed no statistical heterogeneity among the studies (*P* = .84, I^2^ = 0%). Meta-analysis using a fixed-effect model revealed a pronounced reduction in the levels of 8-iso-PGF2α in the experimental group compared with baseline. This difference was statistically significant (mean difference [MD] −29.57 [95% CI −32.65 to −26.49]; *P* < .001) (Fig. 3).

#### 3.3.2. GSH-Px

**Figure 4.**
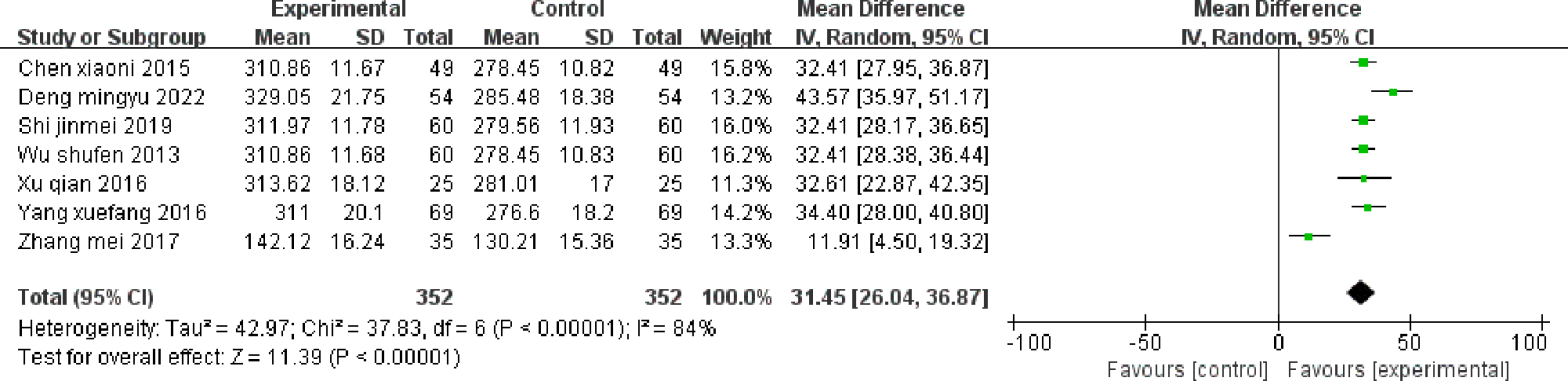
Forest plot of GSH-Px: Forest plot of comparing TCM to promote blood circulation and remove blood stasis combined with laparoscopy versus laparoscopy alone on GSH-Px in endometriosis-related infertility patients. GSH-Px = glutathione peroxidase, TCM = traditional Chinese medicine.

GSH-Px was reported in 7 studies^[19–22,24–26]^; substantial statistical heterogeneity was observed among the studies (*P* < .001; I^2^ = 84%). Meta-analysis using a random-effects model indicated that GSH-Px levels were significantly elevated in the intervention group after treatment compared with the control group, and the difference was statistically significant (MD 31.45 [95% CI 26.04–36.87]; *P* < .001). The results, presented in Figure 4, clearly show a substantial increase in the levels of GSH-Px within the treatment group when juxtaposed with the levels observed in the control group.

#### 3.3.3. RANTES

**Figure 5.**
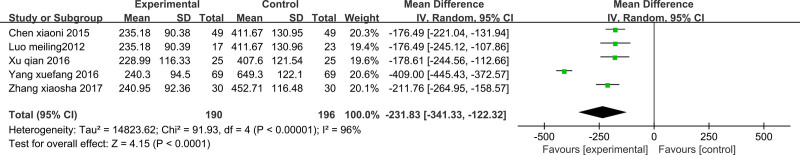
Forest plot of RANTES: Forest plot of comparing TCM to promote blood circulation and remove blood stasis combined with laparoscopy versus laparoscopy alone on RANTES in endometriosis-related infertility patients. RANTES = regulated on activation, normal T cell expressed and secreted, TCM = traditional Chinese medicine.

RANTES was reported in 5 studies^[18,20,25,26,30]^; substantial statistical heterogeneity was observed among the studies (*P* < .001, I^2^ = 96%). Meta-analysis using a random-effects model revealed that posttreatment RANTES levels in the treatment group were substantially reduced compared with those in the control group; the difference was statistically significant (MD −231.83 [95% CI −341.33 to −122.32]; *P* < .001) (Fig. 5).

#### 3.3.4. ROS

**Figure 6.**
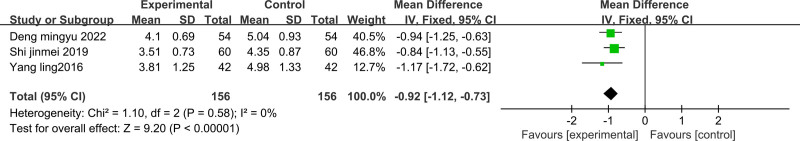
Forest plot of ROS: Forest plot of comparing TCM to promote blood circulation and remove blood stasis combined with laparoscopy versus laparoscopy alone on ROS in endometriosis-related infertility patients. ROS = reactive oxygen species, TCM = traditional Chinese medicine.

ROS was reported in 3 studies^[22–24]^; analysis revealed no statistical heterogeneity among studies (*P* = .58, I² = 0%). Meta-analysis using a fixed-effects model revealed a marked decrease in ROS levels in the group that underwent treatment relative to the control group after the intervention, supported by a statistically significant difference (MD −0.92, [95% CI −1.12 to −0.73]; *P* < .001) (Fig. 6).

#### 3.3.5. Vit E

**Figure 7.**
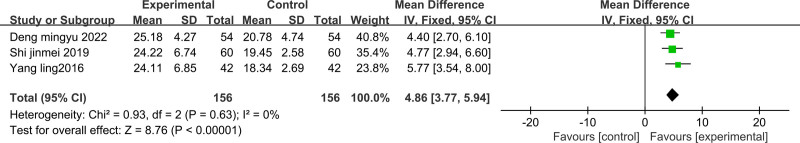
Forest plot of Vit E: Forest plot of comparing TCM to promote blood circulation and remove blood stasis combined with laparoscopy versus laparoscopy alone on Vit E in endometriosis-related infertility patients. TCM = traditional Chinese medicine, Vit E = vitamin E.

Vit E was reported in 3 studies^[22–24]^; analysis revealed no statistical heterogeneity among the studies (*P* = .63, I² = 0%). Meta-analysis performed using a fixed-effects model revealed that Vit E content in the treatment group was considerably elevated compared to that in the control group following treatment (MD 4.86 [95% CI 3.77–5.94]; *P* < .001) (Fig. 7).

#### 3.3.6. MCP-1

**Figure 8.**
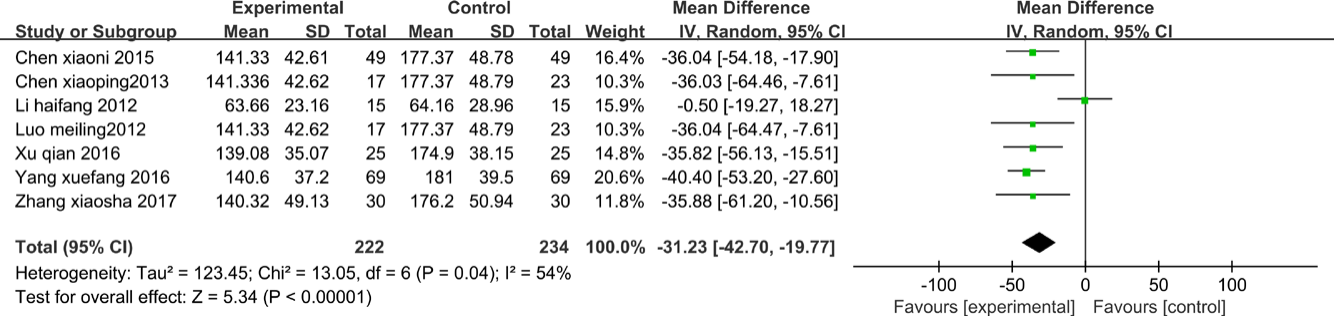
Forest plot of MCP-1: Forest plot of comparing TCM to promote blood circulation and remove blood stasis combined with laparoscopy versus laparoscopy alone on MCP-1 in endometriosis-related infertility patients. MCP-1 = monocyte chemoattractant protein-1, TCM = traditional Chinese medicine.

MCP-1 was reported in 7 studies^[18,20,25,26,28–30]^; analysis revealed no statistical heterogeneity among the studies (*P* = .04, I^2^ = 54%). To account for heterogeneity, meta-analysis was performed using a random-effects model. The findings demonstrated a statistically significant reduction in MCP-1 levels in the treatment group compared with those in the control group posttreatment. The difference was statistically significant (MD −31.23 [95% CI −42.70 to −19.77]; *P* < .001) (Fig. 8).

#### 3.3.7. SOD

**Figure 9.**
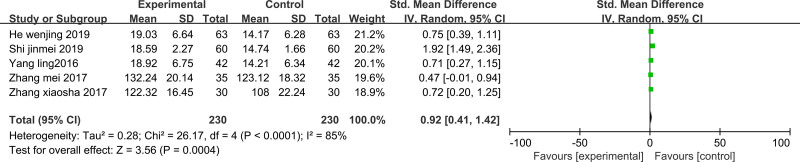
Forest plot of SOD: Forest plot of comparing TCM to promote blood circulation and remove blood stasis combined with laparoscopy versus laparoscopy alone on SOD in endometriosis-related infertility patients. SOD = superoxide dismutase, TCM = traditional Chinese medicine.

SOD was reported in 5 studies^[18,19,22,23,27]^; even with *P* < .001, I^2^ = 85%, signaling substantial heterogeneity, meta-analysis was conducted using a random-effects model. Findings suggested that SOD levels in the treatment group were notably elevated compared to those in the control group following the intervention, and the difference was statistically significant (standardized mean difference 0.92 [95% CI 0.41 to 1.42]; *P* < .001) (Fig. 9).

### 3.4. Sensitivity analyses

#### 3.4.1. 8-ios-PGF2

**Figure 10.**
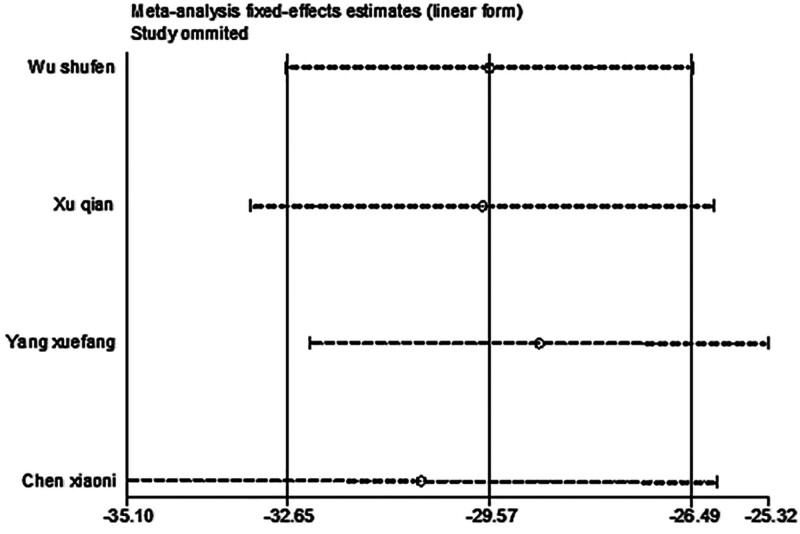
Sensitivity analysis influence plot of 8-ios-PGF2α. 8-ios-PGF2α = 8-isoprostane prostaglandin F2α.

**Figure 11.**
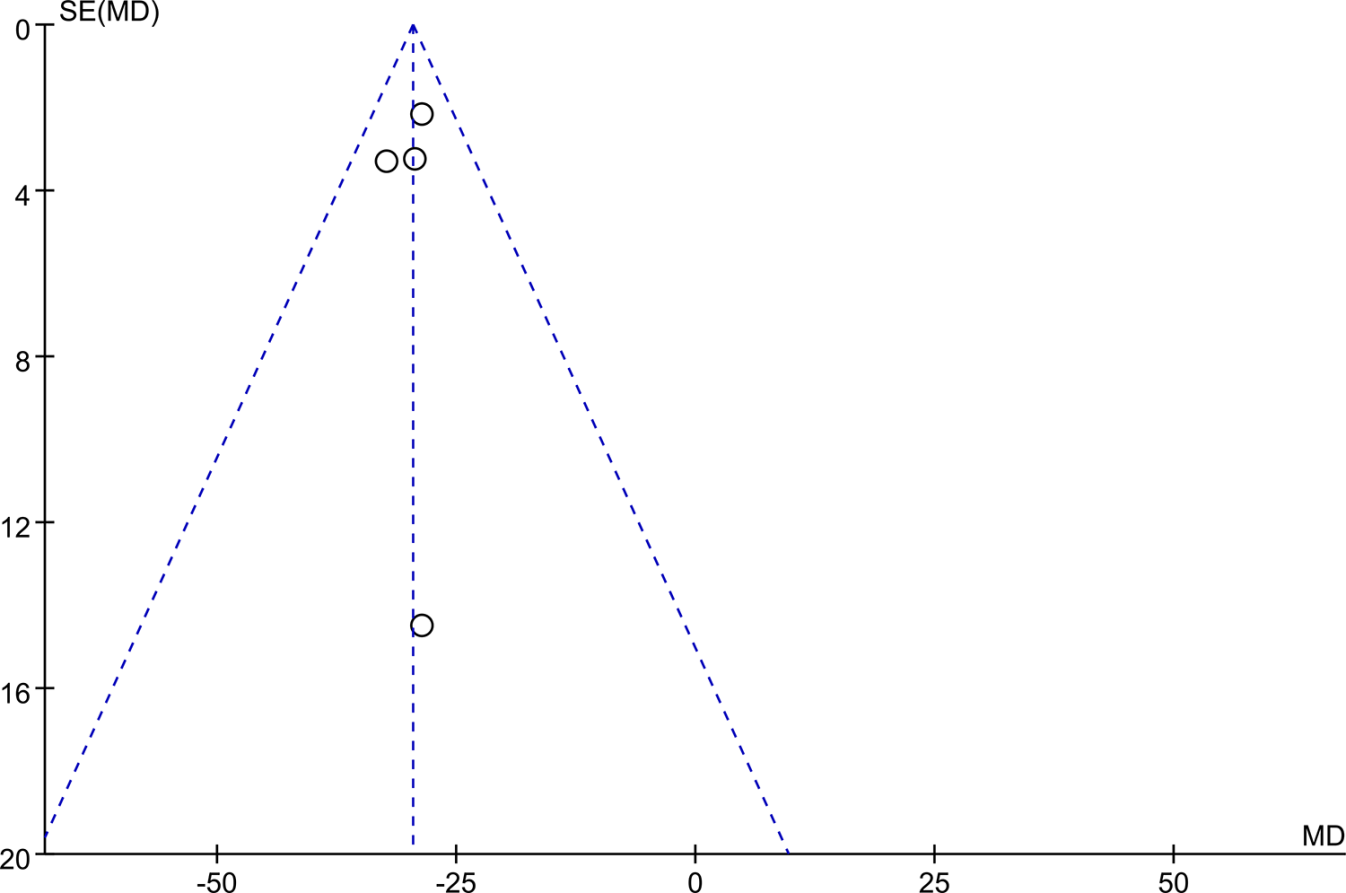
Funnel plot of publication bias of 8-ios-PGF2α content after treatment. 8-ios-PGF2α = 8-isoprostane prostaglandin F2α.

After excluding any single study, a meta-analysis was performed using the remaining literature, and the results were consistent with those of the original meta-analysis, as shown in Figure 10. The symmetry of the funnel plot is shown in Figure 11, and Egger test results (*P* = .767 > .05) indicated no significant publication bias (Table 3). The stability and reliability of the sensitivity analysis for 8-iso-PGF2α further confirms the robustness of the findings.

**Table 3:** Egger test for publication bias of 8-ios-PGF2α content after treatment.

Std_Eff	Coef.	Std. Err.	t	*P*>∣t∣	[95% conf. interval]	
Slope	-28.89303	2.221213	-13.01	.006	-38.45014	-19.33592
Bias	-.2391646	.7071204	-0.34	.767	-3.281658	2.803329

8-ios-PGF2α = 8-isoprostane prostaglandin F2α.

#### 3.4.2. GSH-Px

**Figure 12.**
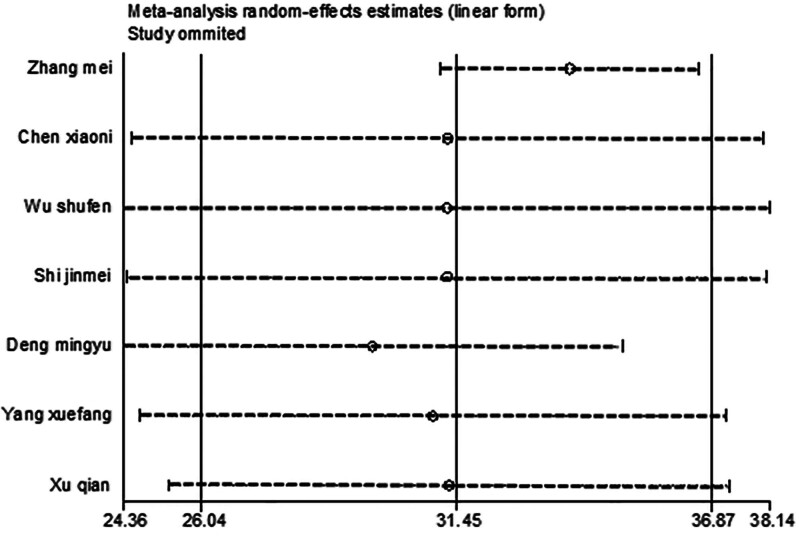
Sensitivity analysis influence plot of GSH-Px. GSH-Px = glutathione peroxidase.

**Figure 13.**
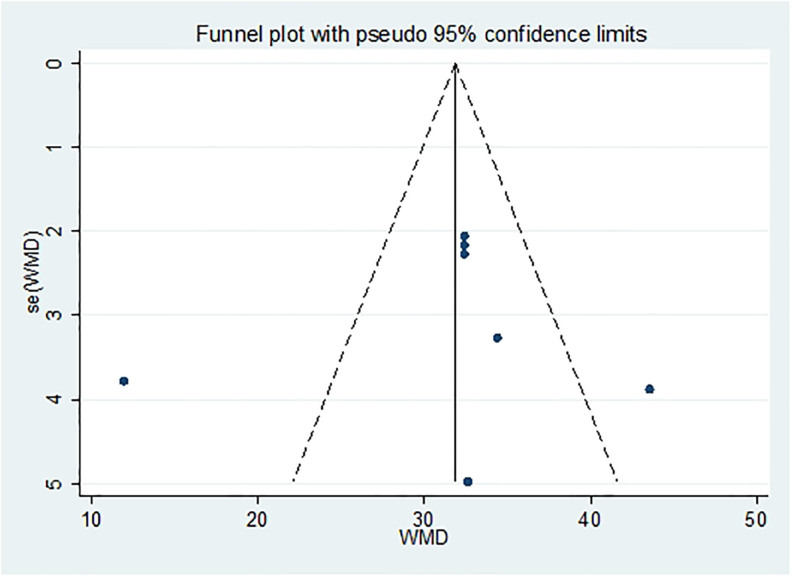
Funnel plot of publication bias of GSH-Px content after treatment. GSH-Px = glutathione peroxidase.

After excluding 1 study at a time (i.e., “leave-one-out” method), meta-analysis was performed on the remaining studies. The results were in agreement with those of the initial meta-analysis (Fig. 12). The symmetry of the funnel plot is shown in Figure 13. The Egger test results, which indicated no significant publication bias (*P* = .796 > .05), are presented in Table 4. The stability and reliability of the sensitivity analysis for GSH-Px further confirmed the robustness of the findings.

**Table 4:** Publication bias of GSH-Px: Egger test for publication bias of GSH-Px content after treatment.

Std_Eff	Coef.	Std. Err.	t	*P*>∣t∣	[95% conf. interval]	
Slope	34.40261	9.725197	3.54	.017	9.403196	59.40203
Bias	-.9592681	3.514937	-0.27	.796	-9.994702	8.076166

GSH-Px = glutathione peroxidase.

#### 3.4.3. RANTES

**Figure 14.**
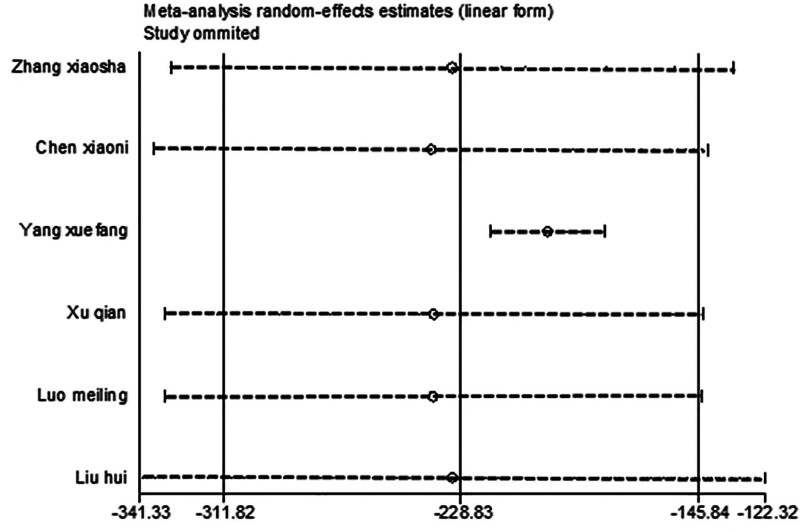
Sensitivity analysis influence plot of RANTES. RANTES = regulated on activation, normal T cell expressed and secreted.

**Figure 15.**
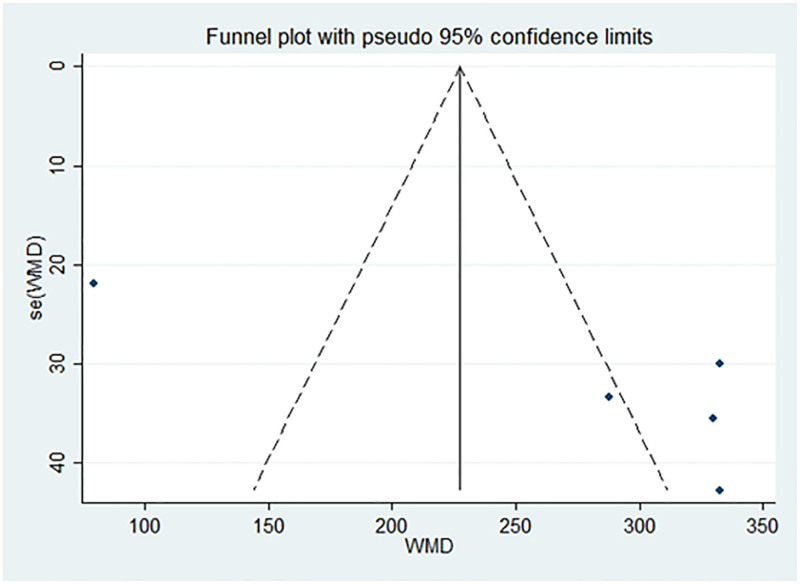
Funnel plot of publication bias of RANTES content after treatment. RANTES = regulated on activation, normal T cell expressed and secreted.

After excluding 1 study at a time (i.e., “leave-one-out” method), meta-analysis was performed on the remaining studies. The results were consistent with those of the original meta-analysis (Fig. 14). The symmetry of the funnel plot is shown in Figure 15. Egger test, which indicated no significant publication bias (*P* = .052 > .05), is detailed in Table 5. The stability and reliability of the sensitivity analysis of RANTES further confirmed the robustness of the findings.

**Table 5:** Publication bias of RANTES: Egger test for publication bias of RANTES content after treatment.

Std_Eff	Coef.	Std. Err.	t	*P*>∣t∣	[95% conf. interval]	
Slope	-213.5303	144.9898	-1.47	.237	-674.9524	247.8918
Bias	14.92266	4.776493	3.12	.052	-0.2782722	30.12359

RANTES = regulated on activation, normal T cell expressed and secreted.

#### 3.4.4. ROS

**Figure 16.**
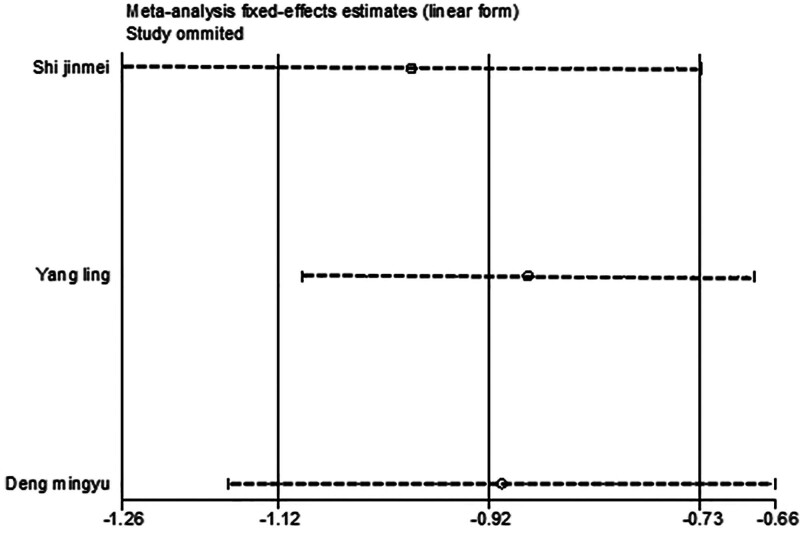
Sensitivity analysis influence plot of ROS. ROS = reactive oxygen species.

**Figure 17.**
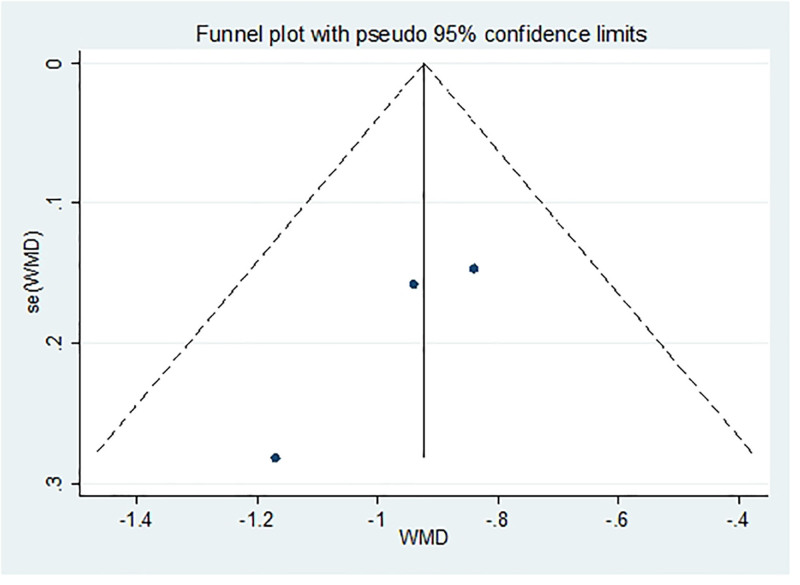
Funnel plot of publication bias of ROS content after treatment. ROS = reactive oxygen species.

After excluding 1 study at a time (i.e., “leave-one-out” method), meta-analysis was performed on the remaining studies. The results were consistent with the results of the initial meta-analysis, as shown in Figure 16. The symmetry of the funnel plot is illustrated in Figure 17. Egger test, which revealed no significant publication bias (*P* = .217 > .05), is shown in Table 6. The stability and reliability of the sensitivity analysis for the ROS further confirmed the robustness of the findings.

**Table 6:** Publication bias of ROS: Egger test for publication bias of ROS content after treatment.

Std_Eff	Coef.	Std. Err.	t	*P*>∣t∣	[95% conf. interval]	
Slope	-.5391927	.1404443	-3.84	.162	-2.323706	1.245321
Bias	-2.278287	.8083972	-2.82	.217	-12.54995	7.993373

ROS = reactive oxygen species.

#### 3.4.5. Vit E

**Figure 18.**
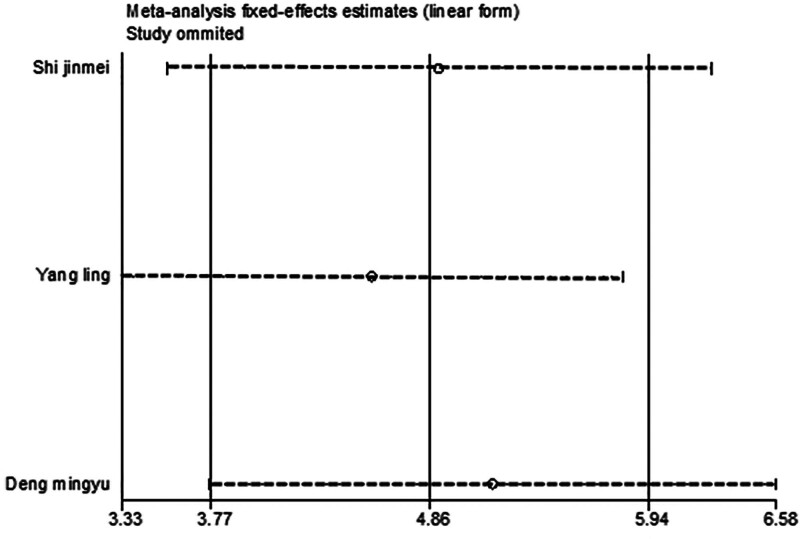
Sensitivity analysis influence plot of Vit E. Vit E = vitamin E.

**Figure 19.**
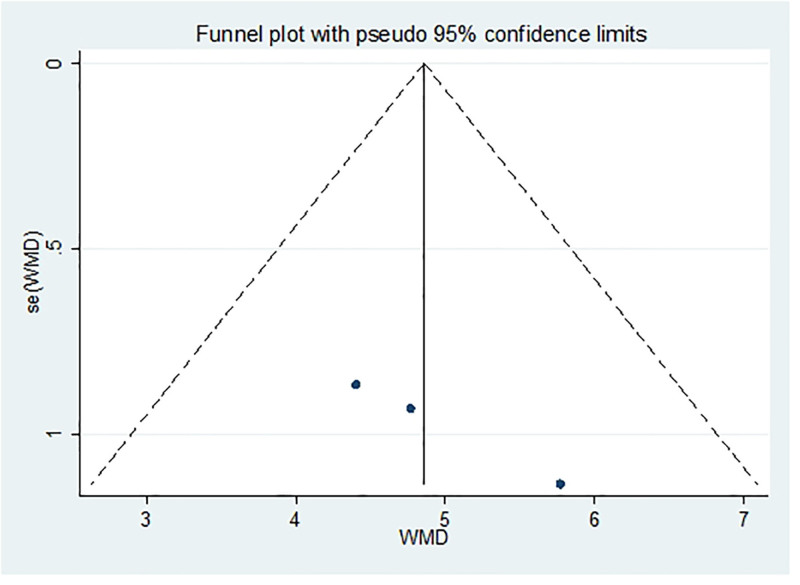
Funnel plot of publication bias of Vit E content after treatment. Vit E = vitamin E.

After excluding 1 study at a time (i.e., “leave-one-out” method), meta-analysis was performed on the remaining studies. The results were consistent with those of the original meta-analysis (Fig. 18). The asymmetry of the funnel plot is presented in Figure 19. The Egger test indicated a potential publication bias (*P* = .025 < .05), prompting the application of the scissor-supplement method. The results revealed minimal variation before and after adjustment, suggesting that the stability and reliability of the sensitivity analysis were maintained despite the asymmetry of the funnel plot (Table 7).

**Table 7:** Publication bias of Vit E: Egger test for publication bias of Vit E content after treatment.

Std_Eff	Coef.	Std. Err.	t	*P*>∣t∣	[95% conf. interval]	
Slope	-.0015023	.1918991	-0.01	.995	-2.439811	2.436807
Bias	5.091231	.1998655	25.47	.025	2.551699	7.630762

Vit E = vitamin E.

#### 3.4.6. MCP-1

**Figure 20.**
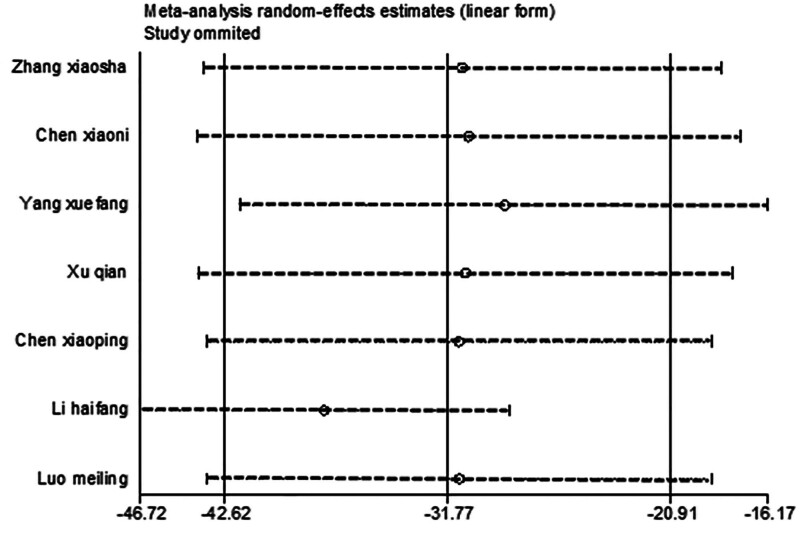
Sensitivity analysis influence plot of MCP-1. MCP-1 = monocyte chemoattractant protein-1.

**Figure 21.**
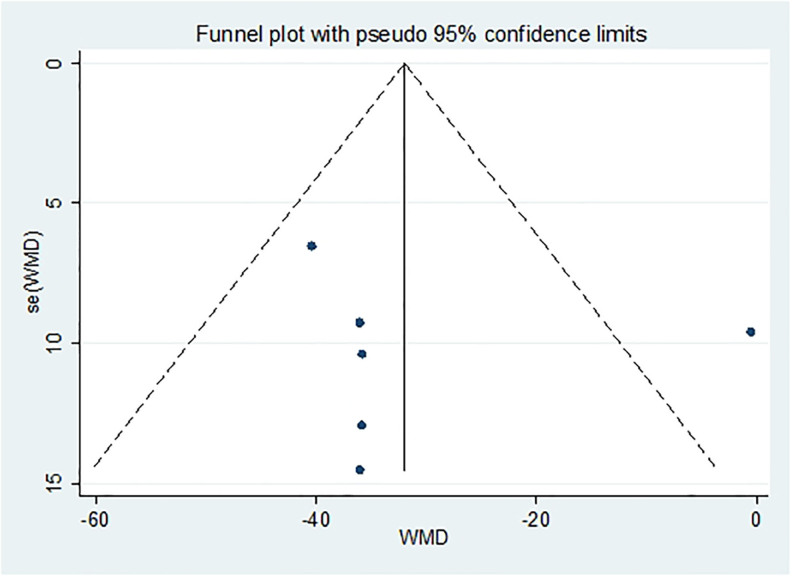
Funnel plot of publication bias of MCP-1 content after treatment. MCP-1 = monocyte chemoattractant protein-1.

After excluding 1 study at a time (i.e., “leave-one-out” method), meta-analysis was performed on the remaining studies. The results were consistent with the original meta-analysis, as shown in Figure 20. The symmetry of the funnel plot is illustrated in Figure 21. The Egger test results, which indicated no significant publication bias (*P* = .794 > .05), are summarized in Table 8. The stability and reliability of the sensitivity analysis of MCP-1 further confirmed the robustness of the findings.

**Table 8:** Publication bias of MCP-1: Egger test for publication bias of MCP-1 content after treatment.

Std_Eff	Coef.	Std. Err.	t	*P*>∣t∣	[95% conf. interval]	
Slope	-37.81276	22.06106	-1.71	.147	-94.52252	18.897
Bias	.6113339	2.219196	0.28	.794	-5.09329	6.315958

MCP-1 = monocyte chemoattractant protein-1.

#### 3.4.7. SOD

**Figure 22.**
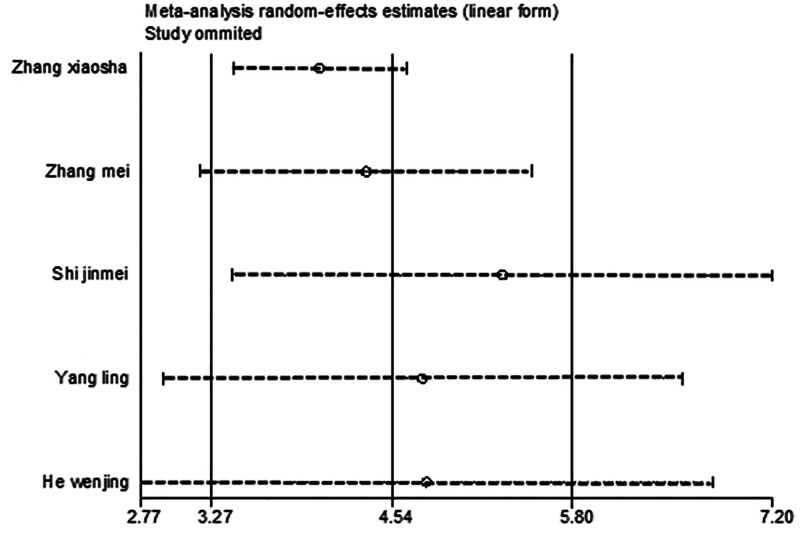
Sensitivity analysis influence plot of SOD. SOD = superoxide dismutase.

**Figure 23.**
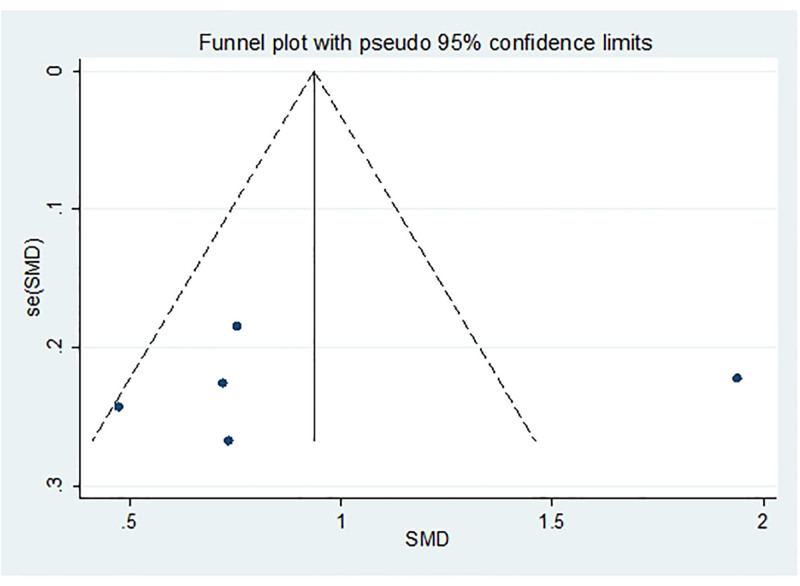
Funnel plot of publication bias of SOD content after treatment. SOD = superoxide dismutase.

After excluding 1 study at a time (i.e., “leave-one-out” method), meta-analysis was performed on the remaining studies. The results were consistent with those of the initial meta-analysis (Fig. 22). The symmetry of the funnel plot is shown in Figure 23. The Egger test results, which indicated no significant publication bias (*P* = .907 > .05), are presented in Table 9. The stability and reliability of the SOD sensitivity analysis further confirmed the robustness of the findings.

**Table 9:** Publication bias of SOD: Egger test for publication bias of SOD content after treatment.

Std_Eff	Coef.	Std. Err.	t	*P*>∣t∣	[95% conf. interval]	
Slope	1.233955	2.379704	0.52	.640	-6.339324	8.807235
Bias	-1.349334	10.66569	-0.13	.907	-35.29233	32.59366

SOD = superoxide dismutase.

### 3.5. Publication bias analysis

Funnel plot and Egger test were used to assess publication bias in the included studies, with a significance threshold of α = 0.05 (i.e., *P* < .05). The funnel plots are presented in Figures 11, 13, 15, 17, 19, 21, and 23, while the results of the Egger test are summarized in Tables 3–9.

## 4. Discussion

The present study meticulously evaluated the effect(s) of combining TCM, which is recognized for its role in enhancing blood flow and resolving blood stasis, with laparoscopic surgery, on the oxidative stress exhibited by patients with infertility due to endometriosis. The investigation revealed that the adjunctive use of TCM to enhance blood flow and resolve stasis, along with laparoscopic surgery, markedly decreased the levels of 8-iso-PGF2α, RANTES, ROS, and MCP-1. Simultaneously, the intervention led to an increase in GSH-Px, Vit E, and SOD levels, thereby improving oxidative stress profiles.

Oxidative stress plays a role in both the development and exacerbation of endometriosis in tandem with inflammatory responses.^[26,30]^ Oxidative stress arises from an imbalance between pro-oxidants, including ROS and reactive nitrogen species, and the availability of antioxidants.^[31,32]^ Furthermore, oxidative stress-induced deoxyribose damage significantly contributes to endometriosis-related infertility.^[33]^

Oxidative stress damages cellular components, including DNA, lipids, and proteins, and may indirectly promote endometriosis by acting as a secondary messenger and activating associated factors and pathways.^[34,35]^ In endometriosis, elevated oxidative stress in the follicular fluid can lead to premature aging of granulosa cells, follicular atresia, meiotic errors in oocytes, maturation issues, and reduced oocyte quality.^[36–38]^ RNA sequencing of oocytes revealed that oxidative stress is a key mechanism of oocyte dysfunction in endometriosis.^[39]^ It also exacerbates endometrial receptivity, affects fertilization and embryo implantation, and has been implicated in infertility.^[40,41]^ Antioxidant therapy improves fertility and reduces adverse pregnancy outcomes in affected women.^[40,41]^ Moderate oxidative stress is necessary for embryo development,^[42]^ and antioxidant defenses, including those involving GSH, SOD, and GPx, are vital for neutralizing ROS and malondialdehyde.^[43,44]^

In our study, we assessed oxidative stress biomarkers critical for antioxidant defense, including SOD, GPx, Vit E, and 8-iso-PGF2α.^[45,46]^ Lower 8-iso-PGF2α levels indicate reduced oxidative stress, potentially linked to endometriosis pathogenesis and ectopic endometrial cell growth.^[47]^ GPx protects the cell membranes from oxidative damage. Chemokines, such as MCP-1 and RANTES, which are key to inflammation and leukocyte infiltration, influence endometriotic lesion development; their reduced levels correlate with suppressed lesion progression.^[48]^ High ROS levels, a result of aerobic metabolism, can lead to oxidative stress and contribute to diseases such as endometriosis.^[34,49,50]^ GPx mitigates oxidative stress in endometriosis-related infertility.^[51]^ Amini et al^[52]^ found that vitE supplementation in patients with endometriosis decreased ROS levels and pelvic discomfort. SOD, crucial for neutralizing free radicals, is often less active in patients with endometriosis, reflecting reduced antioxidant capacity.^[53,54]^

TCM views blood stasis as a key etiological factor in endometriosis,^[55]^ with syndrome differentiation-guiding treatment approaches. The “*Jingyue Book*” describes blood stasis as impure residual blood that causes reversed blood flow and chronic stasis accumulation.^[56]^ Endometriosis is characterized by the inefficient expulsion of impure blood, leading to stasis and potential obstruction of qi and blood flow through the meridians.^[57]^ This can impede the “Chong” and “Ren” meridians, affecting sperm-egg union and contributing to chronic infertility. “*Gynecology of Traditional Chinese Medicine*” categorizes endometriosis into 6 syndrome types, all featuring blood stasis, emphasizing its central role in the pathology. Clinically, the treatment focuses on enhancing blood flow and resolving stasis.

The focus of TCM on enhancing blood flow and resolving stasis has demonstrated benefits in improving circulation and reducing blood viscosity. It also mitigates oxidative stress by facilitating the clearance of byproducts and providing antioxidant properties.^[58]^ By inhibiting endometrial cell adhesion, invasion, proliferation, and neovascularization, TCM may halt disease progression and positively affect reproductive health.^[59]^ Jiaxin et al^[60]^ found that TCM treatment in rats with endometriosis significantly lowered malondialdehyde levels and increased SOD and GPx activity, suggesting enhanced antioxidant defense against oxidative stress.

Studies have confirmed that patients with endometriosis-related infertility have a stasis-type microcirculation, marked by thick, viscous, and hypercoagulable blood, which slows blood flow velocity.^[61]^ Blood circulation enhancement and stasis elimination through TCM have been shown to reduce microcirculatory obstructions and increase blood flow. Additionally, TCM improves the pelvic environment by facilitating peritoneal fluid absorption, restoring fallopian tube function, inhibiting connective tissue growth, and decreasing postoperative adhesions and endometriosis recurrence, thereby potentially enhancing the likelihood of pregnancy.

The present study found that combining TCM with laparoscopic surgery significantly lowered oxidative stress in patients with endometriosis-related infertility. However, it had some limitations, including a lack of detailed randomization methods and an unclear implementation of allocation concealment and blinding among the included studies. Most of the studies were small, single-center trials, which may have introduced bias. In conclusion, the integration of TCM with laparoscopy significantly affected oxidative stress in endometriosis-related infertility. However, given the limitations of the current literature, high-quality, well-designed, RCTs are needed to provide robust evidence for the use of the *Huoxue Huayu* formula combined with laparoscopy to treat endometriosis-related infertility.

## 5. Conclusions

In summary, the activation of blood circulation and removal of blood stasis induced by TCM, when used alongside laparoscopic surgery, demonstrated promise in alleviating oxidative stress in patients and could offer a novel therapeutic strategy to mitigate the pathological effects linked to oxidative stress; however, additional large-scale clinical trials are required to validate these results.

Future research should incorporate large-scale clinical trials to substantiate the efficacy of integrating TCM with laparoscopic interventions for blood circulation enhancement and stasis removal, specifically to mitigate oxidative stress in patients with endometriosis-associated infertility. Future research should also assess the long-term effects of various TCM treatments combined with laparoscopic surgery and their influence on the overall health of patients.

Development and implementation of treatment recommendations include incorporating TCM, designed to facilitate blood circulation and dispel stasis, as a complementary approach to laparoscopic surgery with the aim of optimizing treatment efficacy. Clinicians must closely monitor oxidative stress levels during treatment implementation and adjust the treatment plan based on patient response.

## Author contributions

**Conceptualization:** Shaoqiao Lin.

**Data curation:** Shaoqiao Lin, Shanshan Zhou.

**Methodology:** Shaoqiao Lin.

**Project administration:** Shaoqiao Lin.

**Writing – original draft:** Shaoqiao Lin.

**Writing – review & editing:** Shaoqiao Lin.

## Supplementary Material


