# Supplementary material for: Impact of combining laparoscopy with traditional Chinese medicine on oxidative stress in endometriosis-related infertility: A systematic review and meta-analysis
Source: Medicine (Baltimore). 2025 Apr 11;104(15):e41692. doi: 10.1097/MD.0000000000041692 (PMC11999423; doi:10.1097/MD.0000000000041692)
Supplement: Supplementary file 1 [file medi-104-e41692-s001.doc]

Retrieval Strategies of Different Databases

| Pubmed | | |
| --- | --- | --- |
| 1 | "Endometriosis"[Mesh] | 26362 |
| 2 | ((Endometrioses[Title/Abstract]) OR (Endometrioma[Title/Abstract])) OR (Endometriomas[Title/Abstract]) | 3240 |
| 3 | (((Endometrioses[Title/Abstract]) OR (Endometrioma[Title/Abstract])) OR (Endometriomas[Title/Abstract])) OR ("Endometriosis"[Mesh]) | 27147 |
| 4 | "Infertility"[Mesh] | 75218 |
| 5 | ((((Infertility[Title/Abstract]) OR (Sterility[Title/Abstract])) OR ("Reproductive Sterility"[Title/Abstract])) OR (Subfertility[Title/Abstract])) OR (Sub-Fertility[Title/Abstract]) | 92710 |
| 6 | (((((Infertility[Title/Abstract]) OR (Sterility[Title/Abstract])) OR ("Reproductive Sterility"[Title/Abstract])) OR (Subfertility[Title/Abstract])) OR (Sub-Fertility[Title/Abstract])) OR ("Infertility"[Mesh]) | 125704 |
| 7 | ((Activating blood circulation[Title/Abstract] AND removing blood stasis[Title/Abstract]) OR (huoxuehuayu[Title/Abstract])) OR (huoxue[Title/Abstract]) | 618 |
| 8 | "Laparoscopy"[Mesh] | 123043 |
| 9 | (((((((Laparoscopies[Title/Abstract]) OR (Celioscopy[Title/Abstract])) OR (Celioscopies Peritoneoscopy[Title/Abstract])) OR (Peritoneoscopies[Title/Abstract])) OR (Laparoscopic Surgical Procedure Laparoscopic Surgical Procedures[Title/Abstract])) OR (Laparoscopic Surgery[Title/Abstract])) OR (Laparoscopic Assisted Surgery[Title/Abstract])) OR ("Laparoscopic Assisted Surgeries"[Title/Abstract]) | 27071 |
| 10 | ((((((((Laparoscopies[Title/Abstract]) OR (Celioscopy[Title/Abstract])) OR (Celioscopies Peritoneoscopy[Title/Abstract])) OR (Peritoneoscopies[Title/Abstract])) OR (Laparoscopic Surgical Procedure Laparoscopic Surgical Procedures[Title/Abstract])) OR (Laparoscopic Surgery[Title/Abstract])) OR (Laparoscopic Assisted Surgery[Title/Abstract])) OR ("Laparoscopic Assisted Surgeries"[Title/Abstract])) OR ("Laparoscopy"[Mesh]) | 131443 |
| 11 | ((((((Endometrioses[Title/Abstract]) OR (Endometrioma[Title/Abstract])) OR (Endometriomas[Title/Abstract])) OR ("Endometriosis"[Mesh])) AND ((((((Infertility[Title/Abstract]) OR (Sterility[Title/Abstract])) OR ("Reproductive Sterility"[Title/Abstract])) OR (Subfertility[Title/Abstract])) OR (Sub-Fertility[Title/Abstract])) OR ("Infertility"[Mesh]))) AND (((Activating blood circulation[Title/Abstract] AND removing blood stasis[Title/Abstract]) OR (huoxuehuayu[Title/Abstract])) OR (huoxue[Title/Abstract]))) AND (((((((((Laparoscopies[Title/Abstract]) OR (Celioscopy[Title/Abstract])) OR (Celioscopies Peritoneoscopy[Title/Abstract])) OR (Peritoneoscopies[Title/Abstract])) OR (Laparoscopic Surgical Procedure Laparoscopic Surgical Procedures[Title/Abstract])) OR (Laparoscopic Surgery[Title/Abstract])) OR (Laparoscopic Assisted Surgery[Title/Abstract])) OR ("Laparoscopic Assisted Surgeries"[Title/Abstract])) OR ("Laparoscopy"[Mesh])) | 0 |

| Embase | | |
| --- | --- | --- |
| 1 | 'endometriosis'/exp OR endometriosis | [57481](https://pubmed.ncbi.nlm.nih.gov/?sort=date&term="Endometriosis"%5BMesh%5D) |
| 2 | 'Endometrioses':ab,ti OR 'Endometrioma':ab,ti OR 'Endometriomas':ab,ti | 5206 |
| 3 | #1 OR #2 | 58638 |
| 4 | 'infertility'/exp OR infertility | 220318 |
| 5 | 'infertility':ab,ti OR 'sterility':ab,ti OR 'reproductive sterility':ab,ti OR 'subfertility':ab,ti OR 'sub-fertility':ab,ti | 124141 |
| 6 | #4 OR #5 | 234499 |
| 7 | 'activating blood circulation and removing blood stasis':ab,ti OR 'huoxuehuayu':ab,ti OR 'huoxue':ab,ti | 869 |
| 8 | 'laparoscopic' OR laparoscopic | 262781 |
| 9 | 'laparoscopies':ab,ti OR 'celioscopy':ab,ti OR 'celioscopies':ab,ti OR 'peritoneoscopy':ab,ti OR 'peritoneoscopies':ab,ti OR 'laparoscopic surgical procedure':ab,ti OR 'laparoscopic surgical procedures':ab,ti OR 'laparoscopic surgery':ab,ti OR 'laparoscopic surgeries':ab,ti OR 'laparoscopic assisted surgery':ab,ti OR 'laparoscopic assisted surgeries':ab,ti | 40083 |
| 10 | #8 OR #9 | 264586 |
| 11 | #3 OR #6 OR #10 | 0 |

| Web of Science | | |
| --- | --- | --- |
| 1 | TS=(Endometriosis OR Endometrioses OR Endometrioma OR Endometriomas ) | 46943 |
| 2 | TS=(Infertility OR Sterility OR Reproductive Sterility OR Subfertility OR Sub-Fertility) | 198299 |
| 3 | TS=(Activating blood circulation and removing blood stasis OR huoxuehuayu OR huoxue) | 3252 |
| 4 | TS=(laparoscopic OR Laparoscopies OR Celioscopy OR Celioscopies OR Peritoneoscopy OR Peritoneoscopies OR Laparoscopic Surgical Procedure OR Laparoscopic Surgical Procedures OR Laparoscopic Surgery OR Laparoscopic Surgeries OR Laparoscopic Assisted Surgery OR Laparoscopic Assisted Surgeries) | 217022 |
| 5 | #1 AND #2 AND #3 AND #4 | 1 |

| Cochrane | | |
| --- | --- | --- |
| 1 | Endometriosis | [3427](https://pubmed.ncbi.nlm.nih.gov/?sort=date&term="Endometriosis"%5BMesh%5D) |
| 2 | (Endometrioses):ab,ti,kw OR (Endometrioma):ab,ti,kw OR (Endometriomas):ab,ti,kw | 429 |
| 3 | #1 OR #2 | 3540 |
| 4 | Infertility | 12615 |
| 5 | (Infertility):ab,ti,kw OR (Sterility):ab,ti,kw OR (Reproductive Sterility):ab,ti,kw OR (Subfertility):ab,ti,kw OR (Sub-Fertility):ab,ti,kw | 12146 |
| 6 | #4 OR #5 | 13319 |
| 7 | laparoscopic | 26870 |
| 8 | (Laparoscopies):ab,ti,kw OR ( Celioscopy):ab,ti,kw OR (Celioscopies):ab,ti,kw OR (Peritoneoscopy):ab,ti,kw OR (Peritoneoscopies):ab,ti,kw OR (Laparoscopic Surgical Procedure):ab,ti,kw OR (Laparoscopic Surgical Procedures):ab,ti,kw OR (Laparoscopic Surgery):ab,ti,kw OR (Laparoscopic Surgeries):ab,ti,kw OR (Laparoscopic Assisted Surgery):ab,ti,kw OR (Laparoscopic Assisted Surgeries):ab,ti,kw | 21401 |
| 9 | #7 OR #8 | 26924 |
| 10 | #3 AND #6 AND #9 | 210 |

| CBM | | |
| --- | --- | --- |
| 1 | “endometriosis”[Mesh Terms] | [22549](javascript:historyLink('"子宫内膜异位症"[不加权:扩展]')) |
| 2 | " Endometrioses "[Commonly used fields: intelligence] OR " Endometrial cyst "[ Commonly used fields: intelligence] OR " Endometriosis"[ Commonly used fields: intelligence] OR " Chocolate cyst "[ Commonly used fields: intelligence] OR " Ovarian endometriosis cyst "[ Commonly used fields: intelligence] | [27719](javascript:historyLink('"子宫内膜异位症"[常用字段:智能] OR "子宫内膜异位囊肿"[常用字段:智能] OR "内异症"[常用字段:智能] OR "巧克力囊肿"[常用字段:智能] OR "卵巢子宫内膜异位囊肿"[常用字段:智能]')) |
| 3 | (#2) OR (#1) | [27719](javascript:historyLink('(#2) OR (%231)')) |
| 4 | “Infertility, female”[Mesh Terms] | 16414 |
| 5 | " [infertility](javascript: void(0)) "[Commonly used fields: intelligence] OR " sterility "[ Commonly used fields: intelligence] OR " infertile"[ Commonly used fields: intelligence] OR " female infertility "[ Commonly used fields: intelligence] | 48136 |
| 6 | (#4) OR (#5) | [49136](javascript:historyLink('(#5) OR (%234)')) |
| 7 | “Blood-activating and stasis-removing agent”[Mesh Terms] | [102530](javascript:historyLink('"活血祛瘀剂"[不加权:扩展]')) |
| 8 | " blood-activating and stasis-eliminating method "[Commonly used fields: intelligence] OR " Promoting blood circulation and removing blood stasis "[ Commonly used fields: intelligence] OR " Promoting blood circulation and removing blood stasis "[ Commonly used fields: intelligence] OR " Blood-activating and stasis-removing prescriptions "[ Commonly used fields: intelligence] OR " Chinese herbal medicine for promoting blood circulation and removing blood stasis "[Commonly used fields: intelligence] OR " blood-activating and stasis-removing drugs "[ Commonly used fields: intelligence] OR " improve blood circulation "[ Commonly used fields: intelligence] | [163078](javascript:historyLink('"活血化瘀法"[常用字段:智能] OR "活血化瘀"[常用字段:智能] OR "活血化瘀方剂"[常用字段:智能] OR "活血化瘀中药"[常用字段:智能] OR "活血化瘀药"[常用字段:智能] OR "活血"[常用字段:智能]')) |
| 9 | (#7) OR (#8) | [235456](javascript:historyLink('(#8) OR (%236)')) |
| 10 | ' Laparoscopic examination ' [Unweighted: Extended] | [121873](javascript:historyLink('"腹腔镜检查"[不加权:扩展]')) |
| 11 | "Laparoscopy" [common field: intelligence] OR "laparoscopic surgery" [common field: intelligence] | [184266](javascript:historyLink('"腹腔镜"[常用字段:智能] OR "腹腔镜手术"[常用字段:智能]')) |
|  |  |  |
| 12 | (#10) OR (#11) | [184270](javascript:historyLink('(#11) OR (%2310)')) |
| 13 | (#3) AND (#6) AND (#9) AND (#12) | 82 |

| CNKI | | |
| --- | --- | --- |
| 1 | " Endometrioses "[Commonly used fields: intelligence] OR " Endometrial cyst "[ Commonly used fields: intelligence] OR " Endometriosis"[ Commonly used fields: intelligence] OR " Chocolate cyst "[ Commonly used fields: intelligence] OR " Ovarian endometriosis cyst "[ Commonly used fields: intelligence] OR “endometriosis”[Mesh Terms] | 30479 |
| 2 | " [infertility](javascript: void(0)) "[Commonly used fields: intelligence] OR " sterility "[ Commonly used fields: intelligence] OR " infertile"[ Commonly used fields: intelligence] OR " female infertility "[ Commonly used fields: intelligence] OR “Infertility, female”[Mesh Terms] | 50765 |
| 3 | " blood-activating and stasis-eliminating method "[Commonly used fields: intelligence] OR " Promoting blood circulation and removing blood stasis "[ Commonly used fields: intelligence] OR " Promoting blood circulation and removing blood stasis "[ Commonly used fields: intelligence] OR " Blood-activating and stasis-removing prescriptions "[ Commonly used fields: intelligence] OR " Chinese herbal medicine for promoting blood circulation and removing blood stasis "[Commonly used fields: intelligence] OR " blood-activating and stasis-removing drugs "[ Commonly used fields: intelligence] OR " improve blood circulation "[ Commonly used fields: intelligence] OR “Blood-activating and stasis-removing agent”[Mesh Terms] | [207460](javascript:historyLink('(#8) OR (%236)')) |
| 4 | Laparoscopic examination ' [Unweighted: Extended] OR "Laparoscopy" [common field: intelligence] OR "laparoscopic surgery" [common field: intelligence] | [110](javascript:historyLink('(#11) OR (%2310)')) |

| Wanfang Database | | |
| --- | --- | --- |
| 1 | Theme(" Endometrioses " OR " Endometrial cyst " OR " Endometriosis" OR " Chocolate cyst " OR " Ovarian endometriosis cyst ") AND Theme(" [infertility](javascript: void(0)) " OR " sterility " OR " infertile " OR " female infertility " OR “Infertility, female”[Mesh Terms]) AND Theme(" blood-activating and stasis-eliminating method " OR " Promoting blood circulation and removing blood stasis " OR " Promoting blood circulation and removing blood stasis " OR " Blood-activating and stasis-removing prescriptions " OR " Chinese herbal medicine for promoting blood circulation and removing blood stasis " OR " blood-activating and stasis-removing drugs " OR " improve blood circulation " OR “Blood-activating and stasis-removing agent”[Mesh Terms])AND Theme(“Laparoscopic examination ” OR "Laparoscopy" OR "laparoscopic surgery") | [99](javascript:historyLink('"子宫内膜异位症"[不加权:扩展]')) |

| VIP | | |
| --- | --- | --- |
| 1 | Theme(" Endometrioses " OR " Endometrial cyst " OR " Endometriosis" OR " Chocolate cyst " OR " Ovarian endometriosis cyst ") AND Theme(" [infertility](javascript: void(0)) " OR " sterility " OR " infertile " OR " female infertility " OR “Infertility, female”[Mesh Terms]) AND Theme(" blood-activating and stasis-eliminating method " OR " Promoting blood circulation and removing blood stasis " OR " Promoting blood circulation and removing blood stasis " OR " Blood-activating and stasis-removing prescriptions " OR " Chinese herbal medicine for promoting blood circulation and removing blood stasis " OR " blood-activating and stasis-removing drugs " OR " improve blood circulation " OR “Blood-activating and stasis-removing agent”[Mesh Terms])AND Theme(“Laparoscopic examination ” OR "Laparoscopy" OR "laparoscopic surgery") | 0 |

| Chinese clinical trial registration | | |
| --- | --- | --- |
| 1 | Disease name：endometriosis，research type：prevention, Intervention measure: Laparoscopy | [0](javascript:historyLink('"子宫内膜异位症"[不加权:扩展]')) |
